# Hydatid fluid from *Echinococcus granulosus* induces autophagy in dendritic cells and promotes polyfunctional T-cell responses

**DOI:** 10.3389/fcimb.2024.1334211

**Published:** 2024-05-16

**Authors:** Maia Chop, Camila Ledo, María Celeste Nicolao, Julia Loos, Andrea Cumino, Christian Rodriguez Rodrigues

**Affiliations:** ^1^ Instituto IQUIBIM, Facultad de Ciencias Exactas y Naturales, Universidad Nacional de Mar del Plata (UNMdP), Mar del Plata, Argentina; ^2^ Consejo Nacional de Investigaciones Científicas y Técnicas (CONICET), Argentina; ^3^ Instituto IPROSAM, Facultad de Ciencias Exactas y Naturales, Universidad Nacional de Mar del Plata (UNMdP), Mar del Plata, Argentina

**Keywords:** dendritic cells, hydatid fluid, *Echinococcus granulosus*, autophagy, C-type lectin receptors, T cell responses

## Abstract

Parasites possess remarkable abilities to evade and manipulate the immune response of their hosts. *Echinococcus granulosus* is a parasitic tapeworm that causes cystic echinococcosis in animals and humans. The hydatid fluid released by the parasite is known to contain various immunomodulatory components that manipulate host´s defense mechanism. In this study, we focused on understanding the effect of hydatid fluid on dendritic cells and its impact on autophagy induction and subsequent T cell responses. Initially, we observed a marked downregulation of two C-type lectin receptors in the cell membrane, CLEC9A and CD205 and an increase in lysosomal activity, suggesting an active cellular response to hydatid fluid. Subsequently, we visualized ultrastructural changes in stimulated dendritic cells, revealing the presence of macroautophagy, characterized by the formation of autophagosomes, phagophores, and phagolysosomes in the cell cytoplasm. To further elucidate the underlying molecular mechanisms involved in hydatid fluid-induced autophagy, we analyzed the expression of autophagy-related genes in stimulated dendritic cells. Our results demonstrated a significant upregulation of *beclin-1, atg16l1* and *atg12*, indicating the induction of autophagy machinery in response to hydatid fluid exposure. Additionally, using confocal microscopy, we observed an accumulation of LC3 in dendritic cell autophagosomes, confirming the activation of this catabolic pathway associated with antigen presentation. Finally, to evaluate the functional consequences of hydatid fluid-induced autophagy in DCs, we evaluated cytokine transcription in the splenocytes. Remarkably, a robust polyfunctional T cell response, with inhibition of Th2 profile, is characterized by an increase in the expression of *il-6, il-10, il-12, tnf-α, ifn-γ* and *tgf-β* genes. These findings suggest that hydatid fluid-induced autophagy in dendritic cells plays a crucial role in shaping the subsequent T cell responses, which is important for a better understanding of host-parasite interactions in cystic echinococcosis.

## 1 Introduction

Human echinococcosis is a parasitic disease caused by tapeworms of the *Echinococcus* genus (McManus et al., 2003). Cystic echinococcosis is considered a re-emerging and neglected disease, which not only causes important public health problems but also leads to considerable economic losses in many countries (Moro and Schantz, 2009). Metacestodes are fluid-filled cysts developed by the parasite in the viscera of the intermediate host, such as livestock and occasionally humans. Hydatid fluid (HF) is a clear liquid that has antigenic properties and fills the entire cyst providing nutrition for larval growth (Juyi et al., 2013). HF comprises of more than 150 proteins, most of which are isoforms of different immunodominant lipoproteins, such as EgAgB and EgAg5 (Ahn et al., 2015). In addition, a wide range of host proteins are mainly composed by serum proteins (e.g., β-hemoglobin, albumin and transferrin), antioxidant/xenobiotic enzymes (e.g., selenium-binding protein 1, peroxiredoxin-1), enzymes involved in carbohydrate metabolism (e.g., glutamic-oxalacetic transaminase and glutamic-pyruvic transaminase) and proteins related to immune responses (e.g., immunoglobulins) (Ahn et al., 2015; Zeghir-Bouteldja et al., 2017). In addition to protein content, polysaccharides, lipids, chemical elements (Na2^+^, K^+,^ Ca^2+^, Mg^2+^, Cu^2+^ and Zn^2+^), and biochemical metabolites (e.g., urea, uric acid, glucose, glycogen and free amino acids, including alanine, glycine, and valine) are found in large proportions in HF (Juyi et al., 2013). During the course of the infection, the cyst may rupture, releasing HF into the peritoneal cavity, where *Ehinococcus granulosus*-antigens can be detected by dendritic cells and mount a specific immune response.

Dendritic cells (DCs) are antigen-presenting cells with a unique ability to induce primary immune responses, bridging recognition of pathogenic signals in the periphery to cells of the adaptive immune system (Banchereau et al., 2000). Antigen recognition by different pathogen recognition receptors like C-type lectins in DCs promote migration, cytokine production and antigenic peptide presentation loaded in the Major Histocompatibility Complex (MHC) to T cells (Banchereau and Steinman, 1998). Kanan et al, showed that HF from *E. granulosus* can stimulate the release of IL-12 and IL-6, and the up-regulation of MHC class II and CD86 of DCs (Kanan and Chain, 2006). In contrast, HF-differentiated DCs stimulated with lipopolysaccharide (LPS) showed weaker expression of costimulatory molecules and reduced production of IL-12 and TNF-α (Riganò et al., 2007). We previously observed that HF-stimulated bone marrow derived dendritic cells (BMDCs) induced a slight phenotype maturation with down-modulation of CD40 and unconventional cytokine transcription with IL-6 and IL-10 production (Rodriguez Rodrigues et al., 2021).

Autophagy is a catabolic process that delivers cytoplasmic constituents and damaged organelles into autophagosomes to fuse with lysosomes for the degradation and utilization of self-digestion products (Glick et al., 2010). A substantial body of literature supports the physiological and pathophysiological roles of autophagy, such as starvation adaptation, intracellular protein and organelle clearance, development, cell death and tumor suppression. However, another important function of this process is its role in immunosurveillance, such as the elimination of microorganisms and antigen presentation of endogenous and exogenous proteins loaded on MHC class I and class II molecules, thereby promoting the activation of CD8^+^ and CD4^+^ T cells, respectively (Mizushima, 2005; Jagannath et al., 2009). The excretory/secretory products from *Angiostrongylus cantonensis* induce autophagy via the sonic hedgehog pathway in mouse astrocytes (Chen et al., 2020). In helminths, *Brugia malayi*-stimulated DCs have been shown to increase autophagy in these cells by upregulating phosphorylated Beclin-1 and degrading p62 (Narasimhan et al., 2016). *Heligmosomoides polygyrus* induces inhibition of LC3 expression in macrophages and this process is STAT6 dependent (Su et al., 2012). We have previously reported that HF from *E. granulosus*, significantly promoted LC3 accumulation in autophagosome like-vesicles in DCs compared to purified laminar layer treated or untreated cells (Rodriguez Rodrigues et al., 2021).

T cell responses in *Echinococcus* infection are characterized by early T helper 1 (Th1)-polarized cytokine production, which tries to eliminate the parasite at the initial stages of development and shifts to a predominant Th2 cytokine response in the later chronic stage (Vuitton, 2003). As previously described, HF induced ambiguous IL-6 and IL-10 production in BMDCs and promoted splenocytes proliferation (Rodriguez Rodrigues et al., 2021). Patients with chronic cystic echinococossis generate Th1/Th2 responses accompanied by an increased number of regulatory T cells (Baz et al., 2006). Similarly, *Echinococcus* secreted products induced the release of IL-10 and contributed to the expansion of TGF-β-driven Foxp3^+^ Treg cells (Nono et al., 2012). In the other hand, during the infection course, tissue inflammation characterized by a pro-inflammatory Th17 profile with an expression of RORγt, IL-17, IL-10 and IL-6 significantly increased in the middle stage (day 30-90) in a mouse model of *Echinococcus* infection (Ma et al., 2014; Pang et al., 2014). *E. granulosus* protoscoleces promoted the differentiation of IL-10-producing B cells (B10), IL-17A-producing B cells (B17) and Th17 cells (Pan et al., 2017). We hypothesized that *E. granulosus* strictly depends on cellular communication mechanisms to regulate its development, growth and survival in the host. Likewise, upon recognizing a potential pathogen, the host mounts an immunological response to control the infection which involves changes in its cellular metabolism. A component of the complex composition of HF from *E. granulosus*, such as glycoconjugates or actin-related components, could be the target detected by C-type lectin receptors in dendritic cells. Antigen recognition can then induce the activation of catabolic processes in the cell, which favors the presentation and activation of specific T cells. In the present manuscript, we address how HF from *E. granulosus* induces autophagy, C-type lectin receptors modulation and promotes a polyfunctional T cell response required to trigger an effective anthelminthic immunity.

## 2 Materials and methods

### 2.1 Mice and generation of dendritic cells

Female CF-1 mice (6-8 weeks old), weighing 28-35 g, were provided by the National Health Service and Food Quality (SENASA). Mice were anesthetized with 50 mg/kg ketamine and 5 mg/kg xylazine and sacrificed by cervical dislocation. Bone marrow-derived dendritic cells were obtained by flushing the bone marrow of femurs and tibias as previously described (Lim et al., 2012). Cells were plated at 1x10^6^/ml in RPMI 1640 supplemented with 5% heat-inactivated fetal bovine serum, 100 U/ml penicillin/streptomycin, 10 μg/ml gentamicin and 2 mM L-glutamine (all from Thermo Fisher) and in the presence of 100 ng/ml Flt3L (R&DS Systems) at 37°C in 5% CO_2_ for 6 days. Finally, the DC population was characterized by flow cytometry using fluorescence-conjugated monoclonal antibodies (mAbs) directed against CD11c (HL3), Flt3 (A2F10), CLEC9A (42D2), CD172a (P84), CD45R/B220 (RA3-6B2) and SiglecH (eBiosciences). Approximately 70-80% of the cells were CD11c^+^.

### 2.2 Purification of hydatid fluid from *Echinococcus granulosus*

Hydatid cysts were collected aseptically from three slaughtered infected cattle. The HF was punctured from the cysts, pooled and centrifuged at 2000 × g for 10 min at 4°C. The filtered supernatant was preserved and the protein concentration was determined by absorbance at 280 nm. HF was free of Mycoplasma, as measured by the Mycoplasma PCR detection kit (VenorGeM) and endotoxin was determined by the Limulus Amebocyte Lysate (LAL) method.

### 2.3 Confocal microscopy

For immunofluorescence staining, we followed the protocol described by Rodriguez Rodrigues et al. with minor modifications (Rodriguez Rodrigues et al., 2013). Briefly, BMDCs were harvested after stimulation for 18 h with 200 μg of HF in the presence or absence of 20 nM rapamycin (RAPA) or 100μM chloroquine (CQ) and seeded on alcian blue coverslips for 20 min. The cells were then washed and blocked with PBS-BSA 2% in a wet chamber for 30 min, fixed in 4% PFA for 10 min on ice, washed twice with 0.1 mM glycine in PBS and permeabilized with 0.05% saponin. They were then incubated with mAb anti-LC3-β, (1:100, clone H50, Santa Cruz Biothecnology), or TFEB (1:100, polyclonal, Invitrogen) overnight at 4°C. After washing them in PBS containing 0.1% of Tween-20, the cells were incubated with a goat anti-rabbit secondary antibody conjugated with Alexa 488 (1:400, A-11059) for 1 h at 37°C. The cells were then washed and incubated with 50 ng/ml DAPI (Sigma-Aldrich) to counterstain nuclei. Coverslips were mounted on glass slides using Fluoromount G (Merk). Immunofluorescence images were acquired using an inverted confocal laser scanning microscope (Nikon, Confocal Microscope C1) with a 60 × oil immersion objective. Image analysis was performed using the Fiji software, in which the background was reduced using brightness and contrast adjustments applied to the whole image. Fluorescent intensity graphs were generated using the histogram plugin in the Fiji software. LC3-positive intracellular compartments were quantified manually. TFEB nuclear translocation was analyzed using the Coloc 2 plugin in Fiji software. A total of 20 images per condition from three independent sets of experiments were acquired and analyzed. To evaluate the acidic compartments, live cells with or without treatment were labeled for 4 h with acridine orange or LysoTracker™ Green DND-26 (Invitrogen), according to the manufacturer’s instructions. The cells were then incubated with dye for 30 min at 37°C., then washed in PBS. Finally, acridine orange or LysoTracker median fluorescence intensity (MFI) levels was measured.

### 2.4 Transmission electron microscopy

2.5x10^6^ BMDCs treated *in vitro* with 200 ug HF or 20 nM RAPA were harvested and processed for TEM, as described by (Setum et al., 1993). Briefly, samples were fixed for 10 min in 2.5% glutaraldehyde in 0.2 M cacodylate buffer at pH 7.2 and washed three times with distilled water. The specimens were then dehydrated by sequential incubation in increasing concentrations of ethanol (50-100%) and embedded in Polybed 812 (Polysciences, Warrington, PA, USA). After curing, ultrathin sections were cut, mounted on unsupported 200 mesh copper grids, stained with uranyl acetate (saturated in 1% acetic acid for 1 h) and lead citrate (Reynold’s lead for 5 min) and carbon-coated. Sections were examined in a JEOL 100-CX electron microscope (JEOL Ltd., Tokio Japan).

### 2.5 Quantitative reverse transcription polymerase chain reaction

Total RNA was isolated from DCs or primed splenocytes in all experimental conditions untreated, LPS or HF-treated DCs cultured for 18 h using TRIzol^®^ (Invitrogen), according to the manufacturer’s instructions. The quantity and purity of the isolated RNA were evaluated using a NanoDrop ND‐1000 Spectrophotometer. Total RNA (100 ng-1 µg) was reverse transcribed using random primers and M-MLV RT (Invitrogen). Specific primer pairs for each gene were designed (**Table 1**). Gene expression analysis was performed on a 7500 Real-Time PCR System (Applied Biosystems) using SYBR^®^Green PCR Master Mix for the detection of PCR products (Applied Biosystems). PCR assays were carried out under the following conditions: a holding stage of 95°C (10 min), 40 cycles of 95°C (15 s), 60°C (1 min), and a melting curve stage of 95°C (15s), 60°C (1 min) and 95°C (15 s). The expression levels were evaluated using the 2-ΔΔCt method, and each experiment was performed in duplicate with appropriate non-template controls. The relative amount of each transcript was determined by normalization to GAPDH.

**Table 1:** Nucleotide sequences (5’→3’) of primers used in qPCR.

Gene	Foward	Reverse
***Beclin-1***	GGCCAATAAGATGGGTCTGA	GCTGCACACAGTCCAGAAAA
***Lc3***	CGGCTTCCTGTACATGGTTT	ATGTGGGTGCCTACGTTCTC
***Atg16l1***	GGTGCCGTAGCTTTCTTGAG	ACTGTGTCCAGTGGGGAGAC
***Tfeb***	GCGGCAGAAGAAAGACAATC	CTGCATCCTCCGGATGTAAT
***Tgf-β***	TTGCTTCAGCTCCACAGAGA	TGGTTGTAGAGGGCAAGGAC
***Il-6***	AGTTGCCTTCTTGGGACTGA	TCCACGATTTCCCAGAGAAC
***Tnf-α***	AGCCCCCAGTCTGTATCCTT	CTCCCTTTGCAGAACTCAGG
***Ifn-γ***	ACTGGCAAAAGGATGGTGAC	TGAGCTCATTGAATGCTTGG
***Il-12p35***	CATCGATGAGCTGATGCAGT	CAGATAGCCCATCACCCTGT
***Il-4***	AACGAGGTCACAGGAGAAGG	TCTGCAGCTCCATGAGAACA
***Il-5***	TCAGACTGTGCCATGACTGT	TTCTGTTGGCATGGGGTAGT
***Il-13***	TGGTTCTCTCACTGGCTCTG	CACACTCCATACCATGCTGC
***Il-17***	TCCAGAAGGCCCTCAGACTA	AGCATCTTCTCGACCCTGAA
***Il-10***	CCAAGCCTTATCGGAAATGA	TTTTCACAGGGGAGAAATCG

### 2.6 Flow cytometry

BMDCs were harvested, washed with PBS containing 2% FBS and suspended in a solution containing fluorescein isothiocyanate (FITC), phycoerythrin (PE) and phycoerythrin cyanine 5 (PECy7) -conjugated mAbs at saturating concentrations for 30 min at 4°C, as previously described (Rodriguez Rodrigues et al., 2011). Two additional washes were performed to eliminate not bound antibodies. mAbs directed to CLEC9A (42D2) CD205 (205yekta) and CD11c (HL3) were purchased from eBioscience. In all cases, isotype-matched control antibodies were used and a gate (R1) was defined in the analysis to exclude all non-viable cells and debris based on size and propidium iodide (PI) staining. Analysis of autophagic flow was carried out by washing untreated, HF-stimulated, or CQ-treated BMDCs (100 μM for 1 h) with a permeabilization solution containing PBS 1X, 0.3% triton-X100 and 0.1% BSA. Cells were then incubated with rabbit anti-LC3 primary antibody (1:100, clone H50, Santa Cruz Biotechnology) for 1 h at 4°C, rinsed with permeabilization solution and incubated for 30 min with goat anti-rabbit secondary antibody conjugated with Alexa 488 (1:400, A-11059). In assays of type C receptor functionality, 10 mM of EDTA was used as a Ca2^+^ chelator. The analysis was performed in 20,000 cells acquired using a BD FACS Canto TM II flow cytometer and the FlowJo software. The results are expressed as MFI or percentage of positive cells.

### 2.7 Statistical analysis

Statistical analysis was performed using the R software (https://www.R-project.org). Analysis of variance (ANOVA) and unpaired t-test were used to analyze the set of data and when indicated by significance (*p<0.05, **p<0.01, ***p<0.001, ****p<0.0001), a Tukey *post hoc* test was used to identify pairwise differences.

## 3 Results

### 3.1 Hydatid fluid from *Echinococcus granulosus* induces lysosomal activity in dendritic cells

**Figure 1:**
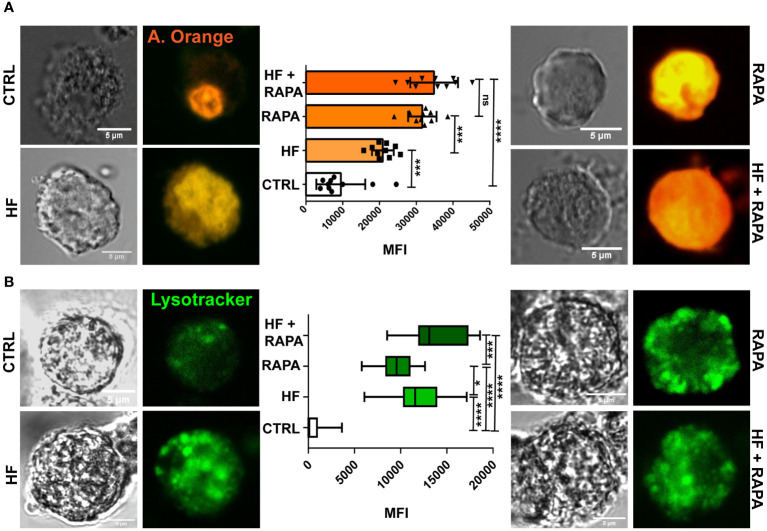
The hydatid fluid of *Echinococcus granulosus* induces the accumulation of lysosomes and a decrease in the pH of these organelles in BMDCs. 1x10^6^/ml BMDCs were stimulated with HF 200 μg from *E. granulosus* for 4 h. Lysosomal acidification in single cells was monitored in HF-stimulated BMDCs using acridine orange and LysoTracker probes and confocal microscopy. The control (CTRL) cells were unstimulated. Rapamycin (20 nM) was added to cells without antigen stimulation (RAPA). HF-stimulated BMDCs alone or in combination with Rapamycin (HF + RAPA) are shown. Scale bar, 5 µm. **(A)** The bar graph shows the acridine orange mean fluorescence intensity ± SEM of different cells in a representative experiment. (One-way ANOVA test ****p<0.0001 and Tukey *post hoc* test ***p<0.001, HF-stimulated cells vs. control; or ****p<0.0001, HF+ RAPA vs. CTRL when indicated). **(B)** The box graph shows the LysoTracker mean fluorescence intensity ± SEM of different cells in a representative experiment. (One-way ANOVA test ****p<0.0001 and Tukey post hoc test ***p<0.001, HF+ RAPA vs. RAPA; ****p<0.0001, HF-stimulated cells, RAPA or HF+ RAPA vs. CTRL, or *p<0.05, HF vs. RAPA when indicated). Three independent experiments were performed.

Bone marrow dendritic cells (BMDCs) were differentiated from hematopoietic progenitor cells in CF-1 mice. Cells were cultured for 6 days in complete medium supplemented with Flt-3-L, as described in the Methods. Prior to studying lysosomal activity in BMDCs, we analyzed cell viability (80-90%) by PI incorporation and cell phenotype by flow cytometry. The purity of CD11c^+^ cells was higher than 75% (range 70–80%) and the expression of CD135, CD172a, CD205, MHC I, MHC II, CD86 and CLEC9A is shown in **Supplementary Figure 1**. We then analyzed whether HF could increase lysosomal accumulation in BMDCs cytoplasm. For this purpose, we used the fluorescent dyes acridine orange and Lysotracker Green, which are weak bases that can cross biological membranes. As shown in **Figure 1A**, the cytoplasm of control cells was slightly labeled with acridine orange, resulting in low lysosomal activity in untreated cells. This probe accumulates in the cell nucleus owing to the presence of nucleic acids that can interacte with this weak base. To complement this assay, LysoTracker Green (**Figure 1B**), a more specific dye for lysosomes and acidic compartments, was used. Both dyes showed statistically significant differences in HF-stimulated BMDCs compared to untreated cells, and accumulated in the cellular cytoplasm. Rapamycin, a specific mTOR inhibitor, has been shown to augment autophagy. Therefore, it was used as a positive control to induce lysosomal activity. The presence of rapamycin under both conditions, with or without HF, strongly increased the signal of the probe in the cell cytoplasm, confirming the induction of typical acidic compartments in the autophagic process. Surprisingly, the combination of HF stimulation and drug treatment showed an additive effect on lysosomal acidification.

### 3.2 Hydatid fluid stimulation induces maturation of autophagic structures in BMDCs revealed by ultrastructural resolution

**Figure 2:**
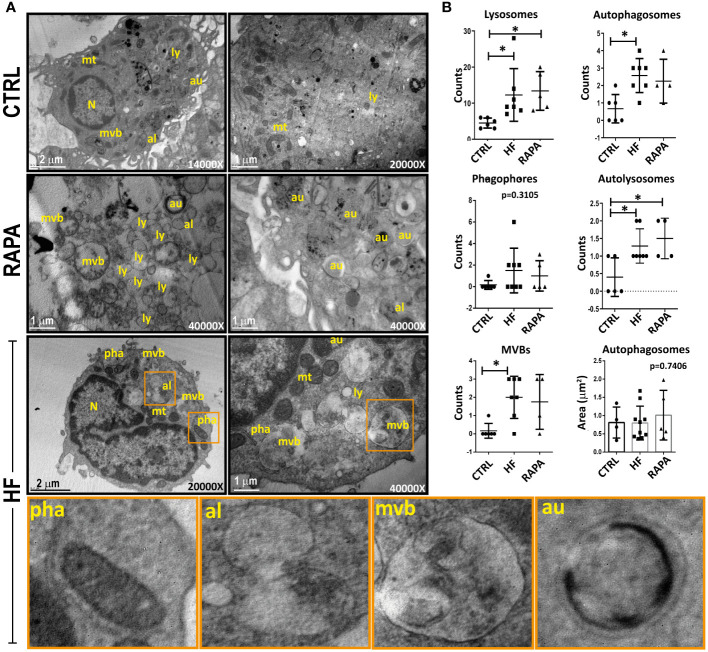
Identification of autophagic structures in hydatid fluid-treated dendritic cells by transmission electron microscopy **(A)** 1x10^6^/ml BMDCs were stimulated with 200 μg of HF from *E. granulosus* for 18 h. Unstimulated cells (CTRL) or 20 nM rapamycin-treated cells (RAPA) were used as negative or positive controls for autophagy induction. Cytoplasms from HF-stimulated BMDCs showed increased vesicularization compared to unstimulated cells. A high-magnification image allows the detection of lysosomes (ly) with its characteristic electron-dense oval structure, autophagosomes (au), a double membrane vesicle with engulfed cytosolic content and autolysosomes (al), a single membrane vesicle. In HF-stimulated BMDCs, phagophores (pha) are cup-shaped, double-membraned precursors that englobe mitochondria (mt). Bars indicate 1-2μm. **(B)** The dotted graph shows the quantification of autophagic structures ± SEM of different images. (one-way ANOVA test and Tukey *post hoc* test *p<0.05, HF-stimulated BMDCs vs. CTRL or RAPA-treated BMDCs vs CTRL when indicated).

To determine whether autophagic activity is altered in BMDCs after exposure to HF from *E. granulosus*, we first examined individual cells using transmission electron microscopy. The appearance of bubble-like vacuoles with double membranes that enclose recognizable cytoplasmic structures represents the gold standard for identifying autophagosomes (Ylä-Anttila et al., 2009). As can be observed in TEM images from control BMDCs, lysosomes (ly) and autophagosomes (au) occasionally appeared in the cell cytoplasm; nevertheless, the number and area of these structures show a cell in a resting state in relation to its vesicular traffic (**Figure 2**). As expected, the presence of rapamycin in the cell culture induced ultrastructural changes in cell membrane redistribution, with a huge accumulation of membrane vacuoles, such as lysosomes, autophagosomes and multivesicular bodies (MVBs). Surprisingly, HF-stimulated BMDCs supported the data from **Figure 1** and showed several morphological features of autophagosome formation, such as isolation-membranes or phagophores (pha) and the formation of double-membraned cisterns in the cell cytoplasm that englobes organelles such as mitochondria (mt) or cellular material (**Figure 2**). In the same way of rapamycin-treated BMDCs, HF induce a markedly accumulation of autophagy structures. Autophagosomes merged with lysosomes to become autolysosomes (al) and partially degraded cargo contents within autolysosomes manifested as irregularly distributed dense masses (**Figure 2**). Other organelles, such as, the endoplasmic reticulum and Golgi apparatus, appeared normal in HF-treated BMDCs. The quantitation of phagophores, autophagosomes, lysosomes and autolysosomes is shown in **Figure 2**. A statistically significant increase in the number of lysosomes, autophagosomes, autolysosomes and multivesicular bodies in the cytoplasm of HF-treated BMDCs compared with that in control cells was detected (p<0.05). No significant differences were observed in the phagophore quantities. The size and area of these structures visibly increased in HF-treated BMDCs compared to those in untreated cells. Taken together, these data and the observation of structures related to the recycling of cellular components indicate that autophagy is induced in BMDCs stimulated with HF from *E. granulosus*.

### 3.3 Hydatid Fluid from *Echinococcus granulosus* induces an upregulation of autophagosome biogenesis genes

We next investigated mRNA expression in BMDCs upon HF stimulation of key autophagy-related genes involved in different functional complexes, in which their proteins are activated and recruited to membranes to initiate autophagy. The analyzed genes were implicated in the initiation of autophagy, such as *beclin-1*, a member of the class III lipid kinase complex I, which produces phosphatidylinositol 3-phosphate. The *atg12* and *atg16l1* genes are part of the subsequent recruitment of the E3-like complex ATG12–ATG5-ATG16L1; the *lc3* gene, a member of the conjugation system, is required for the elongation and maturation of the autophagosome (Pyo et al., 2012). Finally, we evaluated the mRNA expression of the transcription factor EB (TFEB) implicated in the biosynthesis of the lysosome-autophagy pathway. Rapamycin was added to cells as a positive inducer of autophagy.

**Figure 3:**
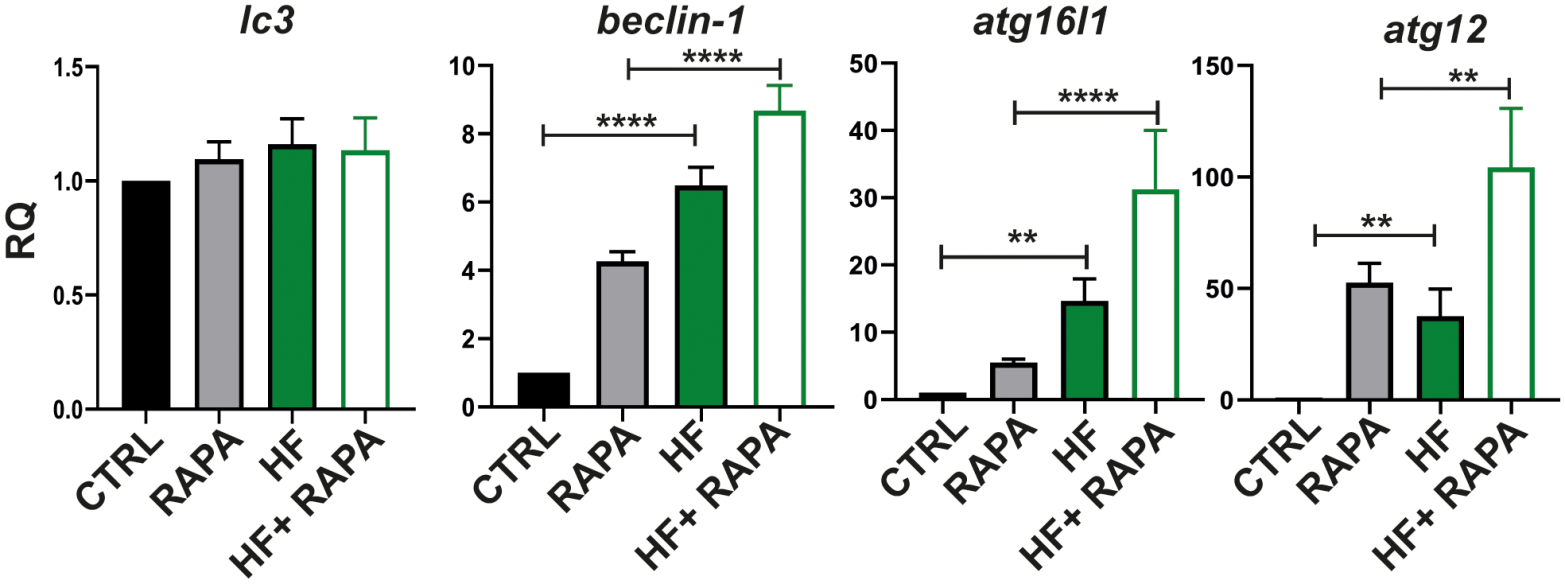
Hydatid fluid from *Echinococcus granulosus* induces gene expression in the autophagy pathway in BMDCs. BMDCs (1×10^6^/ml) were cultured alone (CTRL), treated with 20 nM of Rapamycin (RAPA), with HF stimulation 200μg, or HF in the presence of rapamycin (HF+RAPA). Gene transcription of *lc3, beclin 1, atg16l1 and atg12*, was analyzed 6 h post-stimulation from isolated mRNA by quantitative PCR (relative to the expression of GAPDH mRNA). Results are the mean ± SEM of four experiments performed in duplicate (one-way ANOVA test: ****p<0.0001, **p<0.01, and Tukey post hoc test ****p<0.0001, **p<0.01 for HF-treated BMDCs vs CTRL or HF+RAPA-treated BMDCs vs RAPA-treated BMDCs when is indicated).

As shown in **Figure 3**, HF from *E. granulosus* induced an increase in the transcript levels in BMDCs of *beclin-1* compared to control cells by 5.48 fold (p<0.0001); similarly, HF-treated BMDCs in the presence of rapamycin increased the response of the autophagy inducer by 4.41 fold (p<0.0001) compared to rapamycin control. The member of the atg12 conjugation-complex, *atg16l1 gene*, was upregulated compared to untreated cells by 13.64 fold (p=0.066) and the *atg12* gene was upregulated 36.56 fold (p=0.0234). In the presence of rapamycin, HF-treated BMDCs have shown an increase in *atg16l1* by 25.79 fold (p<.0001) compared to rapamycin-treated BMDCs. In the case of *atg12*, HF stimulation plus the autophagy drug inducer showed an upregulation of 51.80 fold (p=0.0020). In all the genes described above, stimulation plus drug treatment (HF+ RAPA) showed an additive effect on transcriptional upregulation. No significant differences were observed in the *lc3* gene (**Figure 3**) or in the transcription levels of *tfeb* HF-stimulated BMDCs compared with untreated cells (**Supplementary Figure 2**).

### 3.4 Hydatid fluid induces LC3 lipidation of the autophagosome membrane in BMDCs

**Figure 4:**
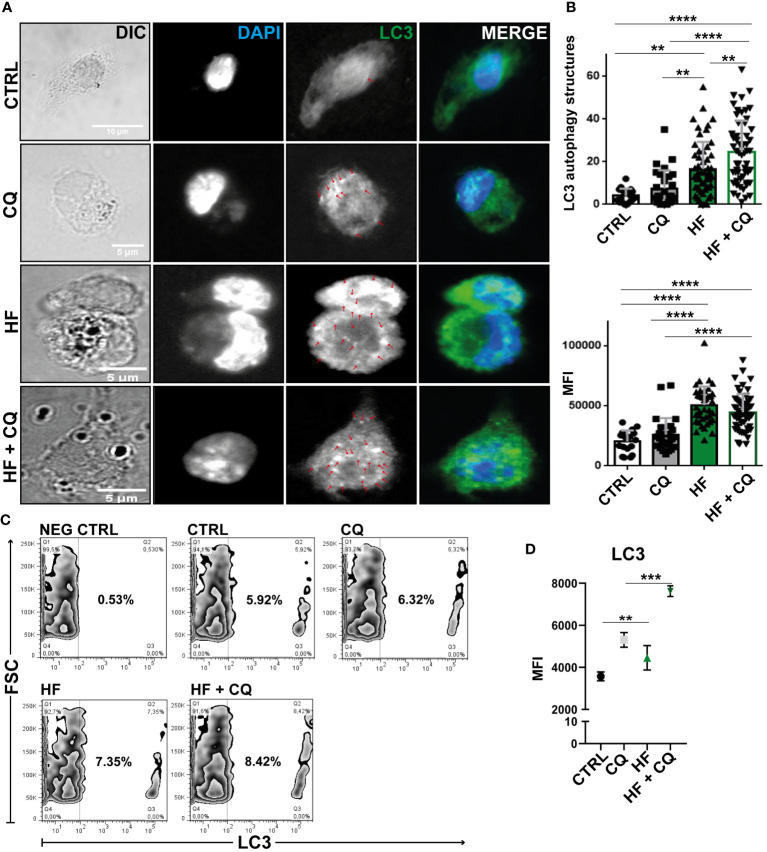
Hydatid fluid from *Echinococcus granulosus* induces accumulation of LC3 in a conjugated form on autophagosome membranes BMDCs (1×10^6^/ml) were cultured without stimulation (CTRL) and treated with 100 μM of chloroquine (CQ), stimulated with 200 μg of HF, or HF in the presence of chloroquine (HF+CQ). **(A)** Confocal images reveal the distribution of LC3 (green) and cell nuclei (blue) in BMDCs. Arrows highlight punctate LC3 structures in the cytoplasm. Scale bar 5 μm **(B)** Bar graphs show the number and MFI of LC3^+^ structures ± SEM of different cells in a representative experiment of three independent experiments (one-way ANOVA test and Tukey’s *post hoc* test, **p<0.01 and ****p<0.0001 HF-stimulated cells vs. CTRL or HF+CQ-treated BMDCs vs. CQ-treated BMDCs when indicated). **(C)** Cell permeabilization allows quantification of LC3 II-containing autophagosome fluorescence using Flow Cytometry. Zebra graphs shows percentage of intracellular LC3^+^ compartments of CTRL, HF-stimulated-BMDCs, treated with CQ alone or in combination with HF stimulation (HF+CQ) for 1 h. Negative control (NEG CTRL) represents the specificity of sample labeling when incubated with the secondary antibody in the absence of the primary anti-lc3. **(D)** Mean intensity fluorescence (MFI) under the same experimental conditions (one-way ANOVA test and Tukey’s post-hoc test, **p<0.01 and ***p<0.001 HF-stimulated cells vs. CTRL or HF+ CQ-treated BMDCs vs. CQ-treated BMDCs when indicated).

Given that BECLIN-1 acts in the initiation of the autophagic process and the transcriptional gene is upregulated, in addition to ultrastructural images showing double-membrane compartments in HF-treated BMDCs, we immunostained the soluble microtubule-associated protein 1A/1B-light chain 3 (LC3) on fixed cells and analyzed them using immunofluorescence confocal microscopy to detect whether it binds tightly to autophagosome membranes upon HF stimulation. As shown in **Figure 4A**, unstimulated BMDCs cultured in complete medium exhibited a dispersed cytoplasmic arrangement of LC3. Chloroquine (CQ), an antimalarial drug, prevents lysosomal function by inhibiting acidification and increasing lysosome pH. This cellular effect discourages degradation and promotes the accumulation of LC3 in autophagosomes (Gonçalves et al., 2019). Therefore, CQ-treated cells were used as a positive control for the autophagocytic process. As expected, 1h of CQ treatment induced an increase in the MFI of LC3 in BMDCs and the number of LC3-positive structures compared with unstimulated cells. In the presence of HF from *E. granulosus* BMDCs, there was a statistically significant increase (p<0.001) in MFI and the number of LC3-positive structures in comparison with unstimulated cells (**Figures 4A, B**). Then, we analyzed the effect of HF stimulation on BMDCs in the presence of CQ. As shown in the confocal images and graphs in **Figure 4**, a statistically significant additive effect was observed in the number of LC3-compartments in comparison to HF-treated BMDCs without the drug (p<0.0001). Finally, we used a quantitative method (Eng et al., 2010) with slight modifications to assess endogenous LC3 and autophagic flux based on cell count. Cell permeability of CQ-treated, HF-stimulated, and untreated cells resulted in the extraction of soluble LC3-I protein and accumulation of the autophagosomal membrane-bound form LC3-II. As shown in **Figure 4C**, stimulation with HF enhanced the percentage of LC3-positive cells compared to that in control cells (CTRL), and this effect was enhanced by CQ. **Figure 4D** shows an increase in the MFI of the intracellular membrane-bound form of LC3. Next, we evaluated the expression of the transcription factor TFEB in BMDCs using confocal microscopy. As shown in **Supplementary Figure 2**, no nuclear translocation was observed following stimulation with HF from *Echinococus granulosus*. This result reflects the lack of transcriptional induction observed, reinforcing that under the experimental conditions used, there was no induction of autophagy through TFEB.

### 3.5 Hydatid fluid recognition by BMDCs induces C-type lectin receptor down-modulation

**Figure 5:**
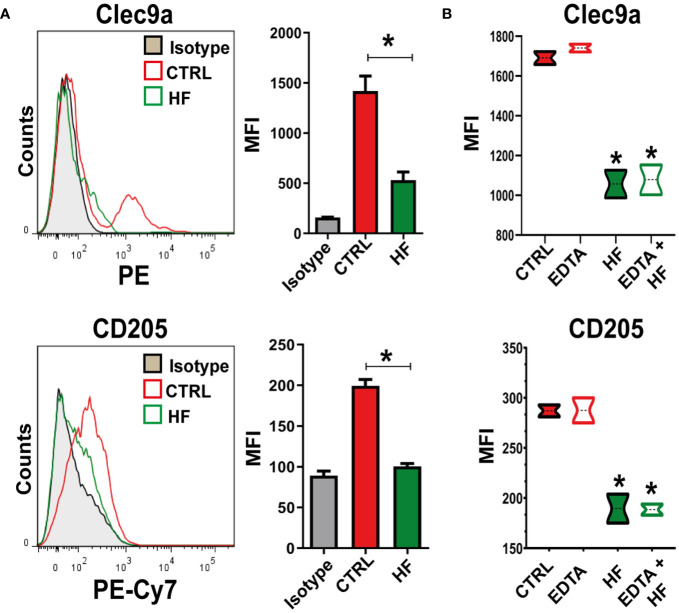
*Echinococcus granulosus* hydatid fluid induces downregulation of CLEC9A and CD205 in BMDCs 1x10^6^/ml BMDCs were stimulated with 200 μg of HF from *E. granulosus* for 18 h. Unstimulated cells (CTRL) were used as negative controls. BMDCs were harvested and the expression of C-type lectins was analyzed by flow cytometry. **(A)** Representative histogram of CLEC9A and CD205 expression at steady state or HF stimulation (n = 4). Bar graphs show the relative mean fluorescence intensity (MFI) of CLEC9A and CD205 in the gate of CD11c^+^ for BMDCs cultured alone (red bars) or under HF stimulation (green bars).U*p<0.05 for CLEC9 A and CD205 in HF-stimulated cells vs. BMDCs cultured alone. **(B)** Bar graphs show the relative MFI of CLEC9A and CD205 in the gate of CD11c^+^ cells for BMDCs cultured alone (red-filled boxes), in the presence of HF (green-filled boxes), or previously treated for 30 min with 10 mM of EDTA (open boxes). Unpaired t-test, *p<0.05 for CLEC9A and CD205 in HF-stimulated cells vs. BMDCs cultured alone or HF-stimulated cells+ EDTA vs. EDTA-treated cells (n=3).

C-type lectin receptors (CLRs) recognize carbohydrate structures such as β-glucans and mannans, which are commonly found in pathogens, such as parasites (Kalantari et al., 2019; Njiri et al., 2020). The responses mediated by CLRs include pathogen binding, phagocytosis, induction of anti-pathogen effector mechanisms, autophagy and production of soluble mediators including cytokines, chemokines and inflammatory lipids (Gringhuis et al., 2007; Brown et al., 2018; Pahari et al., 2020; Del Rio et al., 2022). We next studied CD205 and CLEC9A expression in BMDCs cell membrane upon HF stimulation. As shown in **Figure 5A**, HF induced a statistically significant downregulation of both receptors compared to untreated cells. Next, we evaluated Ca^+2^ dependence on receptors that were down modulated upon HF stimulation. For this purpose, we incubated cells in the presence of 10 mM EDTA for 30 min. As shown in **Figure 5B** the addition of the Ca^+2^ chelator did not induce changes in the expression of CLEC9A and CD205 in the cell membrane of BMDCs after HF exposure.

### 3.6 Hydatid fluid inhibits Th2 profile and potentiates a polyfunctional cytokine response in splenocytes

**Figure 6:**
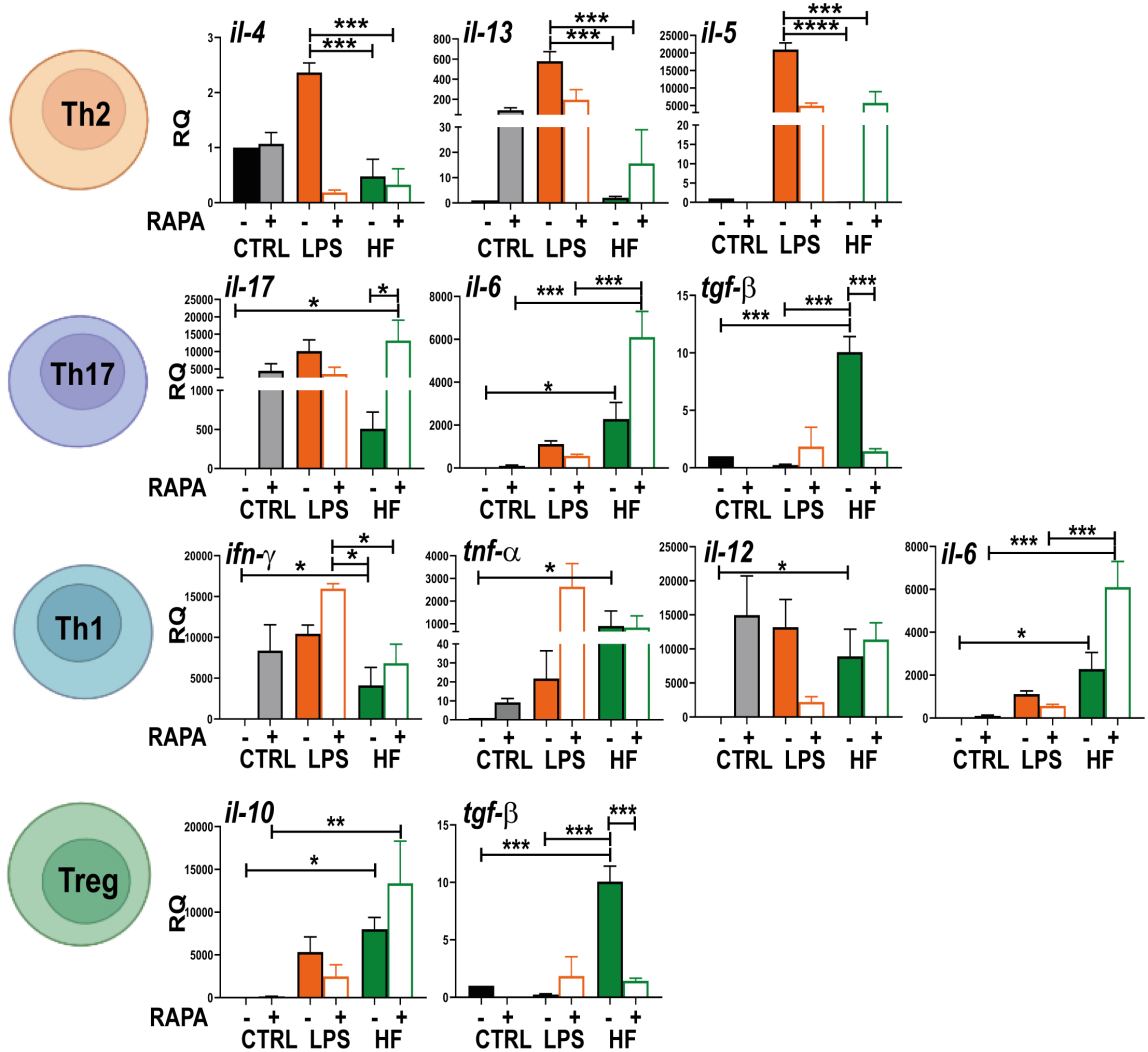
Hydatid fluid from *Echinoccocus granulosus* suppresses Th2 profile and increases cytokines related to Th1, Th17 and Treg responses in splenocytes BMDCs (1× 10^6/^ml) were cultured alone (CTRL), treated with 20 nM rapamycin (RAPA) and stimulated with 100 ng/ml LPS or 200 μg HF in the presence or absence of 20 nM rapamycin for 18 h. Then, they were incubated with resting splenocytes using a DC: splenocyte ratio of 1:4 and cultured in 96-well flat-bottomed plates at a final volume of 200 μl for 4 days. Cells were harvested and gene transcription of different cytokines (*il-4, il-5, il-6, il-10, il-12, il-13, il-17, tnf-α, ifn-γ and tgf-β*) was evaluated from the isolated mRNA by quantitative PCR (relative to the expression of GAPDH mRNA). Bar graphs show the mean ± SEM of three experiments performed in duplicate (one-way ANOVA test ****p<0.0001 and Tukey post hoc test ***p<0.001; **p<0.01, *p<0.05 for splenocytes incubated with HF-treated BMDCs vs. splenocytes incubated with CTRL BMDCs or splenocytes incubated with HF+RAPA-treated BMDCs vs. splenocytes incubated with BMDCs treated with RAPA).

Knowledge of which cytokine pattern is induced after HF stimulation is important to understand the immune response generated to fight and try to clear the infection. Therefore, we evaluated the transcription of genes related to different T response profiles (Th2: IL-4, IL-13 and Il-5; Th17: IL17, IL-6 and TGF-β; Th1: IFN-γ, TNF-α, IL-6 and IL-12; Treg: IL-10 and TGF-β) in co-cultures of splenocytes with BMDCs that were previously stimulated with HF from *E. granulosus*. As shown in **Figure 6**, statistically significant differences were observed in the expression of *il-6, ifn-γ, tnf*-*α*, *il-12, il-10* and *tgf*-*β* (*p<0.05, ***p<0.001 HF vs. control cells) after 6 h of culture. This cytokine induction was enhanced in il-6 and il-10 in the presence of rapamycin (**p<0.01, ***p<0.001 HF + RAPA vs. RAPA). Splenocytes co-cultured with BMDCs previously stimulated with HF also showed a transcriptional increase in il-17 compared to control cells, but this increase was not statistically significant. For cytokines related to a Th2 response profile such as il-4, il-5 and il-13, no gene induction was observed, as was the case with the bacterial lipopolysaccharide (LPS) used as a control. Considering the analyzed transcription profiles, it can be concluded that splenocytes primed with HF-treated BMDCs strongly inhibited the Th2 response and promoted a polyfunctional response with pro-inflammatory profiles such as Th1/Th17, as well as regulatory profiles from a strong induction of genes such as tgf-β and il-10 (**Figure 6**).

## 4 Discussion

In this study, we provide new data on how HF modulates the expression of two C-type lectin receptors, CLEC9A and CD205 in DCs and promotes induction of the catabolic pathway of autophagy. These changes in DCs induce a polyfunctional T-cell immune response against this parasite infection.

CLRs can recognize carbohydrate structures present on the surface of many pathogens in a Ca^+2^-dependent manner (Brown et al., 2018). Previous studies have reported that CLRs are involved in helminth antigen recognition and influence host immune responses (van Die and Cummings, 2010; Hsu et al., 2013; Vázquez-Mendoza et al., 2013; Amri and Touil-Boukoffa, 2015; Barrios et al., 2023). CLEC9A and CD205 are expressed in CD8^+^ BMDCs. These CLR bind damaged and necrotic cells via actin filament recognition and mediates endocytosis and induces pro-inflammatory cytokines via the expression of a cytoplasmic immunoreceptor tyrosine-based activation-like motif (Huysamen et al., 2008; Shrimpton et al., 2009; Sulczewski et al., 2020; Gully et al., 2021). Sequence analysis indicated that both receptors encoded a type II transmembrane protein but lacked the conserved residues involved in calcium ion coordination and carbohydrate-binding found in the classical C-type lectins (Huysamen et al., 2008; Gully et al., 2021). We have described that HF stimulation, through a still-unknown component, probably F-actin or another protein related to necrotic cells, described in proteomic analysis of HF (Zeghir-Bouteldja et al., 2017; Schulz et al., 2018; Canton et al., 2021; dos Santos et al., 2022), induces downregulation of CLEC9A and CD205 in the cell membrane of BMDCs and this effect was not reversed with the use of EDTA.

CLRs have been successfully implicated in restricting infections and inducing autophagy. For example, CLEC4E and TLR4 control *Mycobacterium tuberculosis* growth by increasing lysosome biogenesis, decreasing Il-10 and Il-4 gene expression and enhancing autophagy (Pahari et al., 2020). In eukaryotic cells, recognition of β-glucan motifs on the fungal cell wall by dectin-1 induces LC3 recruitment to phagosomes in macrophages (Tam et al., 2014). In our trials, we observed that stimulation with HF from *E. granulosus* induced an increase in lysosomal biosynthesis. This effect has been reported in intracellular parasitic infections such as *Plasmodium falciparum* and *Trypanosoma cruzi*, where lysomotrophic fluorochromes are used to monitor and mobilize the parasite in acidic compartments (de Carvalho and de Souza, 1989; El Chamy Maluf et al., 2016). Consistent with this increase in lysosomal activity and previous results where we found that HF-stimulated DCs did not activate the mTOR pathway with an induction in global translation, as was observed with the laminar wall (Rodriguez Rodrigues et al., 2021), we detected an increase in cytoplasmic blistering in the HF-stimulated DCs. Autophagic structures, such as phagophores, autophagosomes, and autolysosomes were observed after HF stimulation with ultrastructural resolution. In other helminth products, such as a phosphorylcholine (PC)-containing immunomodulatory glycoprotein secreted by the filarial nematode *Acanthocheilonema viteae*, selective autophagolysosomal degradation has been observed in DCs (Eason et al., 2016). Excretory/secretory products of *Angiostrongylus cantonensis* stimulated autophagosome formation and the expression of autophagy molecules, such as LC3B, Beclin, and p62 in astrocytes (Chen et al., 2020). *Brugia malayi* promoted autophagy in monocytes by showing autophagic vesicles formation, upregulating the mRNA expression of autophagy-related genes *beclin-1, lc3b, atg5, atg7* and increasing LC3B protein levels (Narasimhan et al., 2016).

In addition to the autophagic structures observed in the cell cytoplasm, this study presents the transcriptional promotion of key genes in the autophagic pathway, such as *beclin-1, atg12* or *atg16l1* after HF stimulation. Although an increase in lc3 expression was not observed at the transcriptional level, a significant accumulation of LC3 was detected in autophagosomes at the protein level. This pattern augments with inhibition of lysosomal acidification. The transcription factor EB (TFEB) coordinates lysosomal biogenesis and also regulates autophagy (Settembre et al., 2011). In our assays we did not observe nuclear localization of TFEB after HF stimulation, while TFEB is an important regulator of autophagy, there are other ways to induce this process. One of them involves the activation of AMP-activated protein kinase (AMPK), which inhibits mTOR, and activates the Unc-51-like kinase (ULK1) complex, which is involved in the initiation of autophagy (Egan et al., 2011). Another pathway involves the activation of the class III phosphatidylinositol 3-kinase (PI3K) complex, which includes Beclin 1 and VPS34. Activation of this complex can stimulate the formation of autophagosomes and initiate autophagy independently of TFEB (Matsunaga et al., 2009). Interestingly, it has been observed that HF stimulation plus rapamycin treatment, BMDCs showed an additive effect. This observation was clearly detected in lysosomal acidification, the induction of autophagic gene transcription and the accumulation of LC3^+^ compartments (Rodriguez Rodrigues et al., 2021). The reason behind this stricking effect could be that while rapamycin inhibits mTOR, HF does not; it does not even induce it, but can directly enhance autophagy pathway via activation of the Beclin-1 complex.

Previously, we showed that HF stimulates the upregulation of MHC class II and the production of IL-10 and IL-6 by BMDCs. Similarly, as observed by Kanan et al., DCs stimulated with HF induced the production of IL-12 and IL-6 and the upregulation of MHC class II and CD86 (Kanan and Chain, 2006). In Addition, HF-stimulated BMDCs induced high proliferation of splenocytes in co-culture assays (Rodriguez Rodrigues et al., 2021). Based on our background, we studied the cytokine profile induced by splenocytes primed with BMDCs stimulated with HF. Several studies in patient with cystic echinococcosis and mouse models have shown that T helper 1 (Th1) is potent in the early stages of parasitic infection (Grubor et al., 2017; Rostami-Rad et al., 2018). This response is characterized by the release of IFN-γ after priming DCs with IL-12 (Aliberti et al., 2003). Both cytokines are necessary for the early removal of the parasite. However, long-term infection drives the immune system towards a Th2 response with high levels of IL-4, IL-5, IL-13, and immunosuppressive IL-10 and TGF-β, leading to tolerance (Grubor et al., 2017; Rostami-Rad et al., 2018; Kim et al., 2019). The effects of HF or other *Echinococcus* antigens have been studied *in vitro*. For example, HF induced a mixture of Th1/Th2 cytokines (IL-13, IL-12 and INF-γ in stimulated peripheral blood mononuclear cells (Rigano et al., 2001). HF stimulates T cell lines from patients with inactive cysts to produce IFN-γ (Th1 profile). Conversely, T cell lines derived from patients with active cysts had mixed Th1/Th2 responses (Rigano et al., 2004). In contrast, B cells cultured with excretory-secretory products from *E. granulosus* induce B10 cells, suppress Th1/Th17 responses, and promote the induction of Treg cells (Pan et al., 2017).

Interestingly, unlike the type of T cell response generated in chronic patients with echinococcosis, we found that primed splenocytes with HF-stimulated BMDCs blocked the induction of cytokines related to Th2 profile (*il-4, il5, il-13*). A polyfunctional T response intervened in this regulation, where both pro-inflammatory (*tnf-α, il12, il-6, il-17, ifn-γ*) and anti-inflammatory (*il-10, tgf-β*) cytokines that characterize Th1, Th17 and Treg activation profiles were detected in HF-stimulated BMDCs primed splenocytes compared with untreated cells. Polyfunctional T-cells have been intensively studied in viral, bacterial and parasitic diseases, represent an indicator of protective immunity or disease activity (Duvall et al., 2008; Wilkinson and Wilkinson, 2010; Albareda et al., 2013; Petrone et al., 2015).

In conclusion, we demonstrated the biological and immunomodulatory activities of HF isolated from *E. granulosus*. HF antigens, not yet defined, induce downregulation of C-type lectin receptors, such as CLEC9A and CD205 in BMDCs. This activation is accompanied by catabolic processes that facilitate antigen presentation, T cell proliferation and a development of a polyfunctional T-cell response, reminiscent of the specific anti-parasitic response observed during the early stages of the infection. Additional studies on *in vivo* models are needed for a complete understanding of the role of polyfunctional T-cells subsets in HF stimulation. These results can help to better characterize the immunity of cystic echinococcosis and open the door to new strategies to prevent parasite establishment within the tissues.

## Data availability statement

The raw data supporting the conclusions of this article will be made available by the authors, without undue reservation.

## Ethics statement

The animal study was approved by Animal Care and Use Committee at the Faculty of Exact and Natural Sciences, Mar del Plata University, Argentina (RD544/20). The study was conducted in accordance with the local legislation and institutional requirements.

## Author contributions

MC: Data curation, Formal analysis, Investigation, Methodology, Writing – original draft, Writing – review & editing. CL: Investigation, Methodology, Writing – review & editing. MN: Investigation, Methodology, Writing – review & editing. JL: Investigation, Methodology, Writing – review & editing. AC: Funding acquisition, Investigation, Resources, Writing – review & editing. CRR: Conceptualization, Data curation, Formal analysis, Funding acquisition, Investigation, Methodology, Project administration, Resources, Writing – original draft, Writing – review & editing.

## Supplementary material

Supplementary Figure 1 Viability of BMDCs upon hydatid fluid from E. granulosus stimulation. **(A)** BMDCs (1×106/ml) were stimulated for 18 h with 200 µg of HF from E. granulosus. BMDCs were harvested and cell viability was analyzed by flow cytometry using propidium iodide (PI). The positive control (POS CTRL) for necrosis represents BMDCs cultured in complete medium with 3% DMSO. Unstimulated BMDCs (CTRL) or in presence of hydatid fluid (HF) are shown. Representative histograms of three independent experiments are shown. **(B)** Histograms illustrating the phenotype of BMDCs at a steady state based on the expression of CD11c, MHC class I, MHC class II, CD135, CD172a, CLEC9A, CD205, and CD86 by flow cytometry.

Supplementary Figure 2 The transcription factor TFEB is not involved in the induction of autophagy in BMDCs stimulated with hydatid fluid. BMDCs (1×106/ml) were cultured alone (CTRL), treated with 20nM of rapamycin (RAPA), HF stimulation (200 µg), or HF in the presence of rapamycin (HF + RAPA). Gene transcription of tfeb was revealed 6h post-stimulation from isolated mRNA by quantitative PCR (relative to the expression of GAPDH mRNA). Results are the mean ± SEM of three experiments performed in duplicate. (one-way ANOVA test and Tukey’s post hoc test; *p<0.001; for HF+ RAPA-treated BMDCs vs. untreated cells). **(B)** Nuclear translocation of TFEB was evaluated by confocal microscopy under all experimental conditions.
