# Supplementary figures and images for: Hydatid fluid from Echinococcus granulosus induces autophagy in dendritic cells and promotes polyfunctional T-cell responses
Source: Front Cell Infect Microbiol. 2024 May 16;14:1334211. doi: 10.3389/fcimb.2024.1334211 (PMC11137651; doi:10.3389/fcimb.2024.1334211)

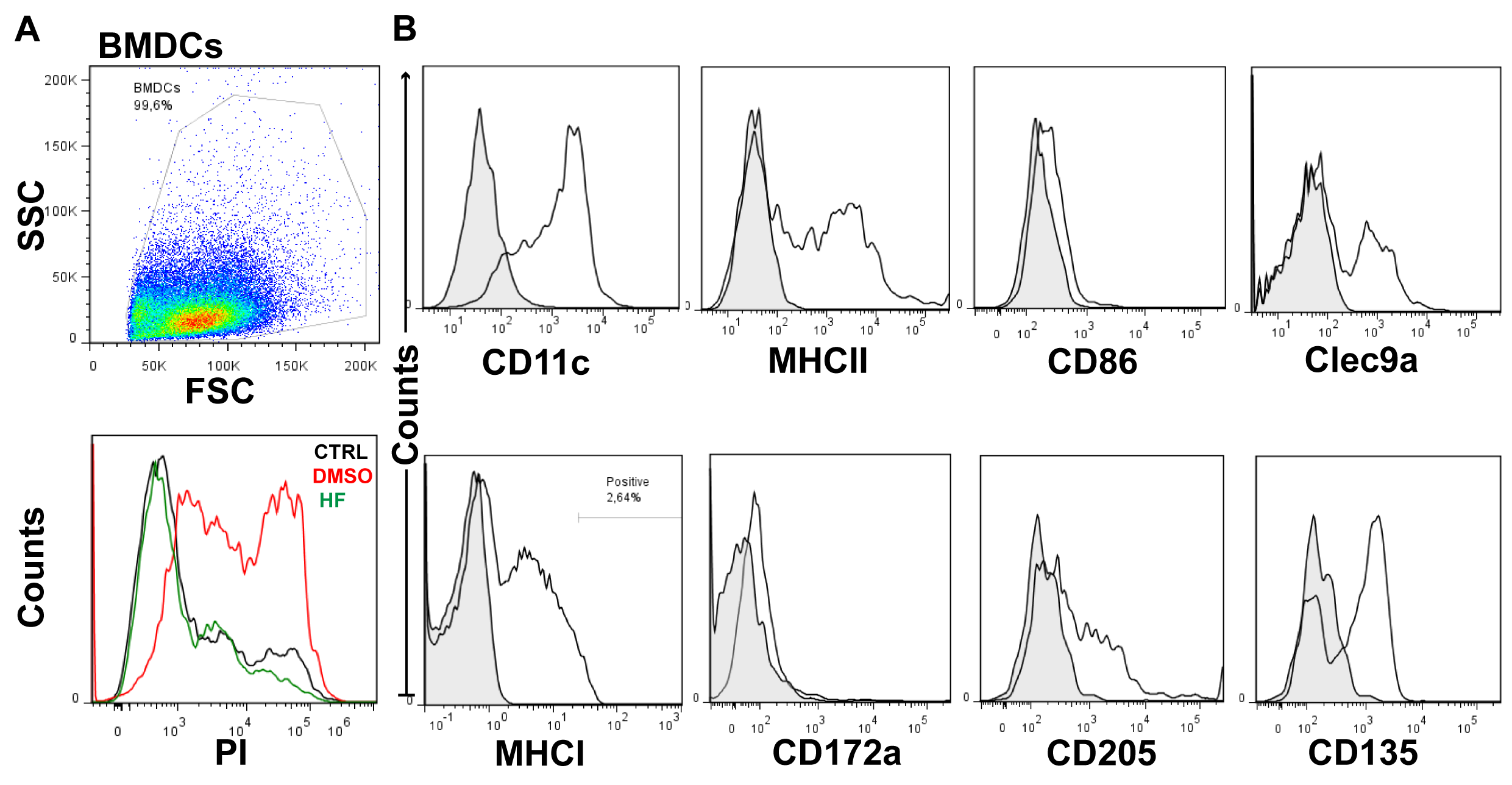

Supplement: Supplementary Figure 1 — Viability of BMDCs upon hydatid fluid from E. granulosus stimulation. (A) BMDCs (1×10^6^/ml) were stimulated for 18 h with 200 µg of HF from E. granulosus. BMDCs were harvested and cell viability was analyzed by flow cytometry using propidium iodide (PI). The positive control (POS CTRL) for necrosis represents BMDCs cultured in complete medium with 3% DMSO. Unstimulated BMDCs (CTRL) or in presence of hydatid fluid (HF) are shown. Representative histograms of three independent experiments are shown. (B) Histograms illustrating the phenotype of BMDCs at a steady state based on the expression of CD11c, MHC class I, MHC class II, CD135, CD172a, CLEC9A, CD205, and CD86 by flow cytometry. [file Image_1.tif]

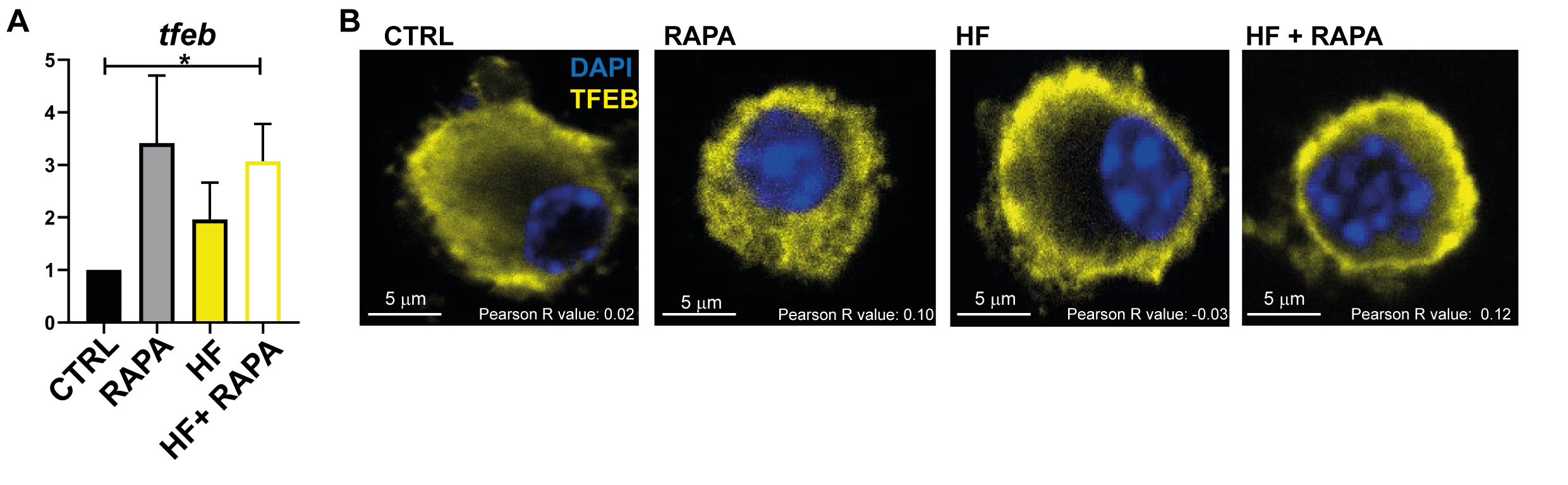

Supplement: Supplementary Figure 2 — The transcription factor TFEB is not involved in the induction of autophagy in BMDCs stimulated with hydatid fluid. BMDCs (1×10^6^/ml) were cultured alone (CTRL), treated with 20nM of rapamycin (RAPA), HF stimulation (200 µg), or HF in the presence of rapamycin (HF + RAPA). Gene transcription of tfeb was revealed 6h post-stimulation from isolated mRNA by quantitative PCR (relative to the expression of GAPDH mRNA). Results are the mean ± SEM of three experiments performed in duplicate. (one-way ANOVA test and Tukey’s post hoc test; *p<0.001; for HF+ RAPA-treated BMDCs vs. untreated cells). (B) Nuclear translocation of TFEB was evaluated by confocal microscopy under all experimental conditions. [file Image_2.tif]
